# Preemptive analgesia with ibuprofen increases anesthetic efficacy in children with severe molar hypomineralization: a triple-blind randomized clinical trial

**DOI:** 10.1590/1678-7757-2021-0538

**Published:** 2022-04-20

**Authors:** Fernanda Vicioni-Marques, Francisco Wanderley Garcia de Paula-Silva, Milena Rodrigues Carvalho, Alexandra Mussolino de Queiroz, Osvaldo de Freitas, Maíra Peres Ferreira Duarte, David John Manton, Fabrício Kitazono de Carvalho

**Affiliations:** 1 Universidade de São Paulo Faculdade de Odontologia de Ribeirão Preto Departamento de Odontopediatria Ribeirão Preto São Paulo Brasil Universidade de São Paulo, Faculdade de Odontologia de Ribeirão Preto, Departamento de Odontopediatria, Ribeirão Preto, São Paulo, Brasil.; 2 Universidade de São Paulo Escola de Ciências Farmacêuticas de Ribeirão Preto São Paulo Departamento de Ciências Farmacêuticas Brasil Universidade de São Paulo, Ribeirão Preto, Escola de Ciências Farmacêuticas de Ribeirão Preto São Paulo, Departamento de Ciências Farmacêuticas, Brasil.; 3 University of Groningen Faculty of Medical Sciences Centrum voor Tandheelkunde en Mondzorgkunde Netherlands University of Groningen, Faculty of Medical Sciences, Centrum voor Tandheelkunde en Mondzorgkunde, UMCG, The Netherlands.

**Keywords:** Molar incisor hypomineralization, Preemptive analgesia, Tooth sensitivity, Dental enamel

## Abstract

Molar incisor hypomineralization (MIH) is often accompanied by dental hypersensitivity and difficulty in achieving effective analgesia.

**Objective:**

This study evaluated the effectiveness of preemptive analgesia in children with severe MIH, post-eruptive enamel breakdown, and hypersensitivity.

**Methodology:**

Ibuprofen (10 mg/kg child weight) or placebo was administered, followed by infiltrative anesthesia and restoration with resin composite. Hypersensitivity was evaluated in five moments. The data were analyzed using the chi-square test, Fisher’s exact test, and t-test.

**Results:**

Preemptive analgesia provided benefits for the treatment of severe cases of MIH, with an increase in the effectiveness of infiltrative anesthesia and improved patient comfort during the restorative procedure.

**Conclusion:**

Preemptive analgesia has shown efficacy in reducing hypersensitivity during restorative dental procedures, evidencing the significance of this study for patients with MIH and hypersensitivity.

## Introduction

Molar incisor hypomineralization (MIH) is a qualitative developmental defect of the enamel that occurs in at least one first permanent molar. Defects can be presented as delimited opacities, ranging from white to yellow and brown, and post-eruptive enamel breakdown (PEB), especially in severe cases.^1^

MIH is one of the most difficult conditions to manage in pediatric dentistry as it is associated with several clinical challenges, including dental hypersensitivity, difficulty in obtaining adequate analgesia/anesthesia, increased risk of carious lesion formation, frequent restoration failure, increased dental anxiety, and impaired esthetics, amongst others.^2-4^

Preemptive analgesia is the use of analgesic therapy prior to a clinical procedure to prevent or reduce trans- and postoperative hypersensitivity; it is often used in third molar extraction and endodontic treatment.^5^ Preoperative analgesia provides several advantages to patients, increasing their comfort during dental procedures and the effectiveness of local anesthesia.^6^ According to patient reports, preemptive analgesia is favorable for the anesthesia of teeth with MIH and increased dental hypersensitivity prior to restorative procedures. However, no clinical studies to date have evaluated the effectiveness of preemptive analgesia in children with MIH.

This triple-blind placebo-controlled randomized clinical trial aimed to evaluate the efficacy of preemptive analgesia and increase the effectiveness of infiltrative anesthesia in children with MIH-associated hypersensitivity and PEB, using ibuprofen (10 mg/kg child weight), which may present side effects such as upset stomach, nausea, vomiting, headache, diarrhea, constipation, dizziness, and drowsiness.

## Methodology

The study was approved by the Research Ethics Committee (CAAE: 10584619.8.0000.5419) and registered on the Clinical Trial platform (NCT03953729). Parents/guardians provided informed consent, and the patients assented. Twenty-three children with MIH aged 6–10 years of both sexes underwent restorative dental treatment. The screening of the children and the restorative treatment were performed by dental clinicians previously trained to manage MIH cases, based on the criteria of Ghanim, et al.^7^ (2015), including only the first permanent molars with hypersensitivity and free of caries lesions.

The analgesia protocol was based on a study by Shantiaee, et al.^8^ (2017), which also used ibuprofen in one of its groups; however, the study analyzed preemptive/preoperative analgesia in patients with irreversible pulpitis. In our study, hypersensitivity was observed in children with MIH, and this specific characteristic is more difficult to determine in the general patient population. No previous study specifically investigated hypersensitivity in MIH patients; therefore, the sample calculation should be based on the acquisition of the general sample as well as the specific pilot study evaluation sample. Accordingly, a total sample of 23 patients who presented with MIH-associated hypersensitivity were included.

All children included in the study had a similar phenotype, with dental hypersensitivity and PEB (score 3),^7^ at least one first permanent molar affected, without caries lesions, and the inclusion criterion was a Wong-Baker scale score above 4 (moderate pain).^9^ The inclusion data indicate the moment T0. Exclusion criteria included intolerance to ibuprofen and developmental defects, including amelogenesis imperfecta and dentinogenesis imperfecta. Medical history and weighing were performed at the time of consultation. Randomization was performed using the “Research Randomizer” (www.randomizer.org), and the numbers obtained were sequentially placed in brown envelopes and retrieved at the time of dental treatment to determine the administration of either the analgesic or placebo.

The analgesic was labeled A; (ibuprofen 100 mg/ml oral suspension; Medley S/A Indústria Farmacêutica, Brazil) and the placebo was labeled B and shared the same characteristics and flavor as the analgesic(formulation without pharmacological effect developed by the Pharmaceutical R&D Laboratory of FCFRP/USP specifically for this study).

The clinician who performed the restorative treatment, the participant, and their guardian did not know which suspension was administered (analgesic or placebo) or who analyzed the data. Only the professional who administered the drug or placebo to the child and an external dental professional knew which was used for each child, in case of any complications during the procedure.

After an estimated action time of 30 min, treatment was initiated. A topical anesthetic gel (Benzocaina 20%, Benzotop^®^, Nova DFL, Rio de Janeiro, Brazil) on a cotton roll was applied to the injection site for 1 min. A standard infiltration anesthetic containing a cartridge (1.8 ml) of 2% mepivacaine with epinephrine 1:100.000 (DFL Indústria e Comércio S/A, Brazil) was applied to each tooth using an aspirating syringe and a 27-gauge 25-mm needle (All Prime^®^, Brazil). The injection of the anesthetic solution had an average duration of 2 min (approximately 1 mL/minute). Subsequently, the tooth was isolated with a rubber dam – with prior infiltrative anesthesia in place, to avoid any discomfort that could be mistaken for dental hypersensitivity due to MIH – only the unsupported enamel was removed, including the tissue already damaged by MIH, followed by cleaning of the cavity with water spray and subsequent drying, application of 37% phosphoric acid for 20 s (AllPrime^®^, Brazil), adhesive placement (Adper^®^ Single Bond, 3M Corp., Brazil), light curing (Emitter H, Schuster^®^, Brazil), and incremental application of resin composite (Filtek Z250 XT, 3M Corp., Brazil).

**Figure 1:**
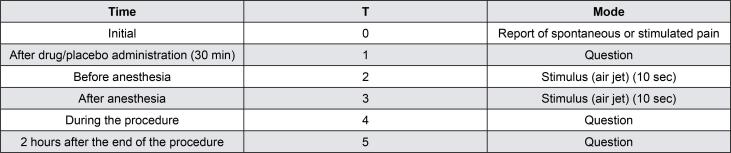
Evaluation times of dental hypersensitivity

**Figure 2:**
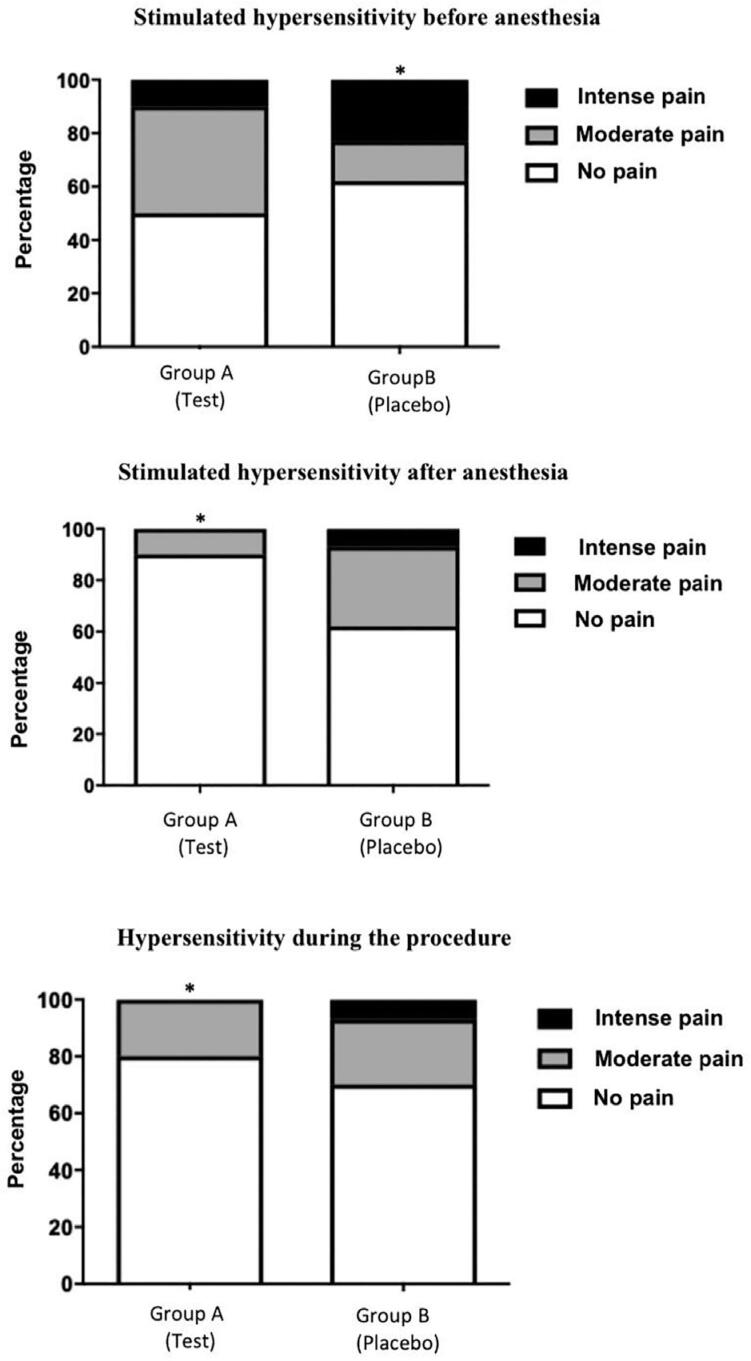
Statistical analysis of hypersensitivity in patients who received analgesic (A) or placebo (B)

Hypersensitivity was measured using the air jet (10 s) test, with the same model of dental units, and the evaluation times are listed in Figure 1. Scores were determined using the Wong-Baker scale.^9^Figure 2 shows all evaluation moments. Statistical analyses included chi-square test, Fisher’s exact test, and t-tests; performed using GraphPad^®^ Prism 8.0 (GraphPad Software, CA, USA). The level of significance was set at 5%.

*statistically significant difference between groups (p<0.05)

## Results

For the evaluation conducted between the groups, a statistical analysis was performed to identify differences in age and gender, and no differences between groups A and B were observed (Table 1).

**Table 1:** Demographic data and distribution of teeth with MIH in the groups

	Group A (Analgesic)	Group B (Placebo)	p
Age	8.4 years (± 1.38)	8.3 years (± 2.06)	p= 0.98
Sex	Male: 70%	Male: 62%	p= 0.29
	Female: 30%	Female: 38%	
Tooth with MIH (n)	16 (2)	16 (5)	p = 0.02
	26 (5)	26 (2)	
	36 (2)	36 (1)	
	46 (1)	46 (5)	

Hypersensitivity, a characteristic factor of MIH and emphasized in this study, was verified 30 min after drug or placebo administration for each group (T1), resulting in a significant difference between groups, which highlights the effectiveness of the analgesic (group A) (p=0.0001).

**Figure 3:**
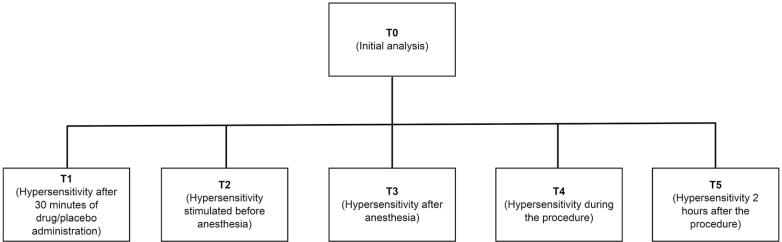
Evaluation time points

The intensity of hypersensitivity triggered by an air stream following ibuprofen administration but before anesthesia (T2) was higher in group B than in group A (p=0.0001). The intensity of hypersensitivity triggered after anesthesia (T3) in group A was lower than that in group B (p=0.0001), and the hypersensitivity during the procedure (T4) was lower in group A than in group B (p<0.0195). T5 showed no significant difference regarding hypersensitivity assessed 2 h after the procedure with respect to the administration of medication or placebo (p=0,8850) (Figure 3).

## Discussion

This is the first clinical trial to examine the efficacy of preemptive analgesia in relieving hypersensitivity in MIH molars with PEB. Hypersensitivity associated with MIH-affected molars is most likely due to the ultrastructural characteristics of decreased enamel mineral density and increased porosity, which provide bacterial access to the underlying dentin, promoting inflammation, increased innervation and hypervascularity of the pulp, and subsequent hyperreactivity to hypersensitivity stimuli.^10^ These changes in the pulp can potentially lead to inadequate local analgesia.^11-13^

Dental clinicians may erroneously increase the amount of anesthetic used in cases of MIH, intending to provide an effective measure for hypersensitivity; however, the anesthetic dose should be considered in the context of the child’s weight and age. No anesthetic has shown superiority over another in these specific cases.^14-16^

Rolim, et al.^17^ (2020) highlighted the importance of restoring teeth with MIH-associated hypersensitivity. The authors found that self-reported dental hypersensitivity and anxiety levels decreased after treatment. In our study, dental hypersensitivity was reduced both during and after the procedure, providing increased comfort to the patient and ostensibly reducing anxiety, which contribute to an improved technique, thus increasing the chances of clinical success.

Some children may present moderate dental hypersensitivity without the presence of a stimulus, as was the case at the time of screening (T0), where no stimulus was present, despite children’s reports of pain as shown by the Wong-Baker scale. This finding demonstrates that children with MIH may have a history of spontaneous pain associated with teeth that have a defect. A hypersensitivity score of 4 was considered in the inclusion criteria since, according to the scale used, it is the minimum limit for moderate pain, which was indicated in this study.

A successful intervention for MIH-associated hypersensitivity positively affects children’s oral health and quality of life, as confirmed by Fütterer, et al.^18^ (2020). However, during these interventions, children may report some operative discomfort due to inadequate anesthesia. The present study confirmed the efficacy of preemptive analgesia in these situations, allowing for greater analgesic potential using infiltrative anesthesia.

Since this is the first clinical trial to evaluate the efficacy of preemptive analgesia in relieving dental hypersensitivity in children with MIH, no direct comparisons can be made between current and previous data. This study has some limitations, such as the broad age range of the participants (6–10 years). However, some studies that evaluated the efficacy of other therapeutic agents in relieving dental hypersensitivity used even broader age ranges.^19-21^ Additionally, we carried out the evaluations using different methods and scales since we considered that the placebo and/or analgesic effect could be well distributed among the groups. Alternatively, we could have assessed the analgesic effect for longer periods; we had to consider, however, that this may have required evaluation of patients by phone or keeping the family in the dental office after the procedure, which could present some difficulty. Further studies using different protocols and pharmacological agents, such as nitrous oxide, acupuncture, or different analgesics, should be performed to provide additional alternatives to treat MIH-affected children.

The administration of pre-emptive ibuprofen analgesia successfully increased anesthetic efficacy and decreased trans-operative hypersensitivity associated with restorative treatment of MIH-affected permanent molars with PEB.

MIH is still under research, that is, there is no vast comparison in the literature as it is a topic in the field of dentistry that has been recently addressed. However, this situation is of enormous clinical concern, especially considering the hypersensitivity suffered by these patients. Thus, this study, for the first time, described and analyzed which measures under preemptive analgesia can be taken to relieve hypersensitivity in children with MIH.
