# ERRATUM

**DOI:** 10.1590/1678-7757-2022er001

**Published:** 2022-05-09

Due to a publishing error the article: “Preemptive analgesia with ibuprofen increases anesthetic efficacy in children with severe molar: a triple blind randomized clinical trial”, published at Journal of Applied Oral Science 2022;30:e20210538 was printed with the following error:

Where it reads:

Title: Preemptive analgesia with ibuprofen increases anesthetic efficacy in children with severe molar: a triple blind randomized clinical trial

The sentence should read:

Title: Preemptive analgesia with ibuprofen increases anesthetic efficacy in children with severe molar hypomineralization: a triple-blind randomized clinical trial

